# "The Hospital" Nursing Section

**Published:** 1906-09-15

**Authors:** 


###  The Hospital.

### Nursing Section.

Contributions for "The Hospital," should be addressed to the Editor, "The Hospital" Nursing Section, 28 & 29 Southampton Street, Strand, London, W.C.

No. 1,043.—Vol. XL. SATURDAY, SEPTEMBER 15, 1906.

### Notes on News from the Nursing World.

### AN INDICTMENT OF THE MIDWIVES ACT.

Last week we called attention to the fact that ignorant midwives are still abroad. To-day we publish a letter from an inspector of midwives who affirms that it is almost impossible to insist upon the rules of the Central Board being carried out, and that, in her opinion, the working of the Act in country districts is impracticable unless the poor are to be left without any help at all at the most critical time in their lives. Our correspondent, in whose district some seventy or eighty registered midwives are practising, as well as a score of unregistered women, supports her views by citations from her own experience. Out of all the registered and unregistered women whom she inspects, she states that there are only four who have received any training whatever, that quite half are between sixty and seventy-five years of age, and that many can neither read nor write. For the rest, the examples which she gives afford convincing proof that Devonshire is not the only county in which ignorant midwives are still abroad. As to the reason of this deplorable condition of affairs, it is summed up in the sentence that "there is not enough work to tempt a trained midwife to enter the field, and certainly not enough to enable her to live." But surely in this sentence also is written the failure of the Act for the Registration of Midwives to achieve the purpose for which it was enacted.

### THE PERILS OF NURSING CLASSES.

It has been decided by the Axminster Higher Education Committee to proceed with the formation of a nursing class for the town. We regard attempts to impart a smattering of nursing as more likely to do harm than good. It is true that first-aid lectures may sometimes be of practical value, and we gave an instance some time ago of a woman who was able to make timely use of the knowledge obtained by her daughter and communicated to her second-hand. But more recently we mentioned a case of the development of pneumonia by a child ill with measles, which was due to the opening of a window in very cold weather by a person who had attended classes on "Home Nursing." We fear that if nursing classes of the kind projected at Axminster are encouraged, the dangers of a little knowledge will more frequently be illustrated in an unpleasant and perhaps serious manner.

### DISMISSAL OF A SUPERINTENDENT NURSE.

The Brentford District Council have dismissed the superintendent nurse at the Isolation Hospital under their control, and censured other members of the staff, for allowing an infant suffering from scarlet fever to be put in a ward where there was an outbreak of measles. The patient appears to have been recovering from scarlet fever, but contracted measles, from which he died. There is no possible justification of the course pursued, and the Brentford Council are to be congratulated upon the opportunity which is now afforded them of securing the services of a more capable staff.

### CANDIDATES FOR A COTTAGE HOSPITAL MATRONSHIP.

Whatever may be the difficulties of cottage hospital matrons, it seems that the desire to obtain one of these posts is very keen. We understand that no fewer that a hundred and twenty-five applications were received for the office of matron of Abingdon Cottage Hospital, to which an appointment was made on Friday last. There are ten beds in the institution, and, in addition to the matron, a district nurse and a single probationer are on the staff.

### WHAT IS A PROBATIONER?

An interesting point was debated at the last meeting of the Romsey Guardians. The Local Government Board having been asked to sanction the appointment of two young women as probationers for the Romsey Poor-law Infirmary, have declined to do so "unless they were to receive training," and suggested that they should be called assistant nurses. The Guardians replied that they would receive training at the hands of the medical officer and the head nurse, but the Local Government Board merely rejoined that their suggestion should be adopted. In discussing this matter, the Guardians took up an attitude of hostility to the Local Government Board, and the Vice-Chairman, in particular, counselled resistance. The issue is of more than local importance, because it raises the question, What is a Probationer? The Local Government Board obviously do not consider that anyone is entitled to be so regarded who is not undergoing the recognised course of training. While sympathising with this view, we do not think that, because the name is used against their wish, they can surcharge the salaries of the officials whose appointment, under another description, they admit to be necessary.

### A COMPROMISE AT LYNN.

The King's Lynn Guardians have further considered the question of the nursing staff, and have arrived at a decision which must be regarded as a compromise. Their own Committee having endorsed the opinion of the Rev. A. H. Hayes and the medical officer to the workhouse, that there should be two nurses on duty during the night in two blocks of buildings, they could not do otherwise than assent to the conclusion; but the Committee also reported against the augmentation of the nursing staff, and the second night nurse will thus be obtained by the reduction of the nurses on day duty. Mr. Hayes, while expressing his satisfaction with the determination to have two night nurses always on duty, protested against the reduction of the day staff, and we doubt whether the compromise agreed to in order to save expense will either be found to work out to the advantage of the patients in the infirmary, or tend to promote the efficiency of those who are responsible for their care.

### ARMY SISTERS IN CAMP.

There was an interesting spectacle in Brisbane during July, when the nurses of the Australian Army Service went for the first time into camp, and the public had the opportunity of inspecting a field hospital complete in all its details. It consisted of six tortoise tents, used as hospital wards, each with ten beds, an operating tent, and a number of bell tents for the *personnel* of the corps. The surgical and medical equipments were displayed in a separate tent, and an imposing array of instruments, bandages, surgical and nursing appliances were on view. The nurses present, who consisted of the superintendent, one matron, and eleven sisters, wore also for the first time the new uniform—grey dresses and red capes—of the Service, which was very conspicuous at the church parade on Sunday.

### THE TRAINING SCHOOLS RECOGNISED BY THE MIDWIVES BOARD.

At the instance of Mr. Helme, M.P., a return showing the names of institutions approved as training schools for midwives by the Central Midwives Board and the number of midwifery cases treated by each during the twelve months previous to the Board's approval, has been issued as a Parliamentary paper. We notice that of London institutions the British Lying-in Hospital treated 484 external and 665 internal; the City of London Lying-in Hospital 611 internal and 2,601 external; Clapham Maternity Hospital 413 internal and 870 external; the General Lying-in Hospital 614 internal and 2,059 external; Guy's Trained Nurses' Institutions 40 internal and 1,216 external; the London Hospital 99 internal and 748 external; Plaistow Maternity Charity 4,025 external; Queen Charlotte's Lying-in Hospital 1,560 internal and 1,969 external; and Shoreditch Union Infirmary 114 external. In the provinces there were treated at Birmingham Union Infirmary 200 external; Brighton and Hove Hospital for Women 15 internal, 1,022 external; Hull Lying-in Charity 769 internal; Ladies' Charity and Lying-in Hospital, Liverpool, 322 internal and 1,847 external; St. Mary's Hospitals, Manchester, 263 internal and 530 external; Nottingham Infirmary 94 internal; Sheffield, Jessop Hospital for Women, 296 internal and 800 external; and West Ham Union Infirmary 180 internal. In Wales 83 cases were treated by Cardiff Union Infirmary internal, 90 by the Cardiff branch of Queen Victoria's Jubilee Nurses' Institute internal; and 165 by the Newport and Monmouthshire Hospital external. The Maternity Hospital, Glasgow, treated 660 cases internal and 1,750 external; Edinburgh Royal Maternity Hospital 395 internal and 520 external; Dundee Maternity Hospital 297 internal and 745 external. In Ireland, Belfast Incorporated Maternity Hospital treated 326 internal and 312 external; Belfast Union Maternity Hospital 366 internal; the Rotunda Hospital, Dublin, 1,676 internal and 2,007 external; the National Maternity Hospital, Dublin, 609 internal and 925 external; the Camp Hospital, Dublin, 586 internal and 1,859 external. At Motlibai Hospital, Bombay, 783 cases were treated internal, and at Eden Hospital, Calcutta, 600 internal.

### PIANOS FOR POOR-LAW NURSES.

The Stepney Guardians have received from the Local Government Board official sanction for the purchase of a piano for use in the Nurses' Home at the Poplar and Stepney Sick Asylum. We think that the Stepney Guardians did well to ask for permission to buy the piano, and that the Local Government Board acted wisely in not withholding it. The cost of a piano for the use of a large staff is, even from a ratepayer's point of view, trifling, and there is no doubt that concessions of this kind tend to render nurses more contented, and therefore more valuable.

### AN APPEAL FROM WESTERN CHINA.

Bishop Cassels has written from Tao-ning, Western China, a letter on behalf of missionary work in China, in which he says:—"It is many years since we have been so destitute of any medical help in this station and district as we are just now. Even though we had no doctor, we nearly always had some qualified nurse and someone who could undertake dispensary work; but now we are absolutely without any help of this kind, and the workers are so busily occupied that in case of any sickness requiring nursing we should be in a very difficult position." If any fully-qualified nurse who is considering the great needs of the heathen and of those engaged in missionary efforts, would like to hear further of the condition of work in Western China, Miss E. Van Sommes, Hon. Secretary of Bishop Cassels' Committee of Help, Cuffnels, Weighbridge, will be pleased to meet or to correspond with her.

### NURSING ASSOCIATION OR COTTAGE HOSPITAL?

The ratepayers of Okehampton met on Saturday night under the presidency of the Mayor to consider the desirability of establishing a cottage hospital. It was stated that there were funds amounting to about £400 available for the purpose, and that an annual income from non-ecclesiastical charities of over £80 a year could be devoted to its maintenance, while a site had been offered for the hospital. The opinion was expressed that a cottage hospital was not only unnecessary but unworkable, the town being favourably situated for sending patients to the South Devon and East Cornwall Hospital at Plymouth, or the Devon and Exeter Hospital at Exeter, if necessary, for treatment. The proposal therefore for the establishment of a cottage hospital obtained but few supporters, whereas a subsequent proposal "to expend the income of the charities in the employment of two domestic nurses for the homes of *bona fide* working-classes, the present Nursing Association being asked to co-operate," was practically carried unanimously. It is hoped that the site offered for the erection of a hospital will be given for the erection of a nurses' home.

### AMATEUR COOKS.

Three cookery pupils celebrated obtaining their certificates by giving a supper—which they cooked themselves—at St. Ives, East Melbourne, to the President and four other members of the Council of the Royal Victoria Trained Nurses' Association. Six courses, admirably cooked, followed in their proper order, and great surprise was expressed at the proficiency attained in six months. The fun, however, was not confined to the supper-room, much as the guests enjoyed the novel entertainment; it was in the kitchen that the excitement waxed fast and furious. Here the rapidity with which the courses were finished threatened at first to disturb the amateur cooks considerably, and one of them was heard to declare that "no human beings had ever eaten so quickly." At 9.30, however, the pupils walked into the supper-room with unruffled faces and their uniforms as clean and trim as if they had just been setting out to Sunday morning service.

### DEATH OF A MATRON.

On Monday last week Miss Alice A. Smith, matron of the General Infirmary, Hertford, passed peacefully away at her home in Paignton, Devon, after a painful and trying illness of more than four months' duration. She was trained at the London Hospital, and was afterwards sister in that institution for eight or nine years. Subsequently, she was home sister at Poplar Accident Hospital for two years—a post which she resigned in order to take up that of matron of the General Infirmary, Hertford. She will be keenly missed by all her nurses, past and present, to whom she was not only a much-appreciated matron, but a sympathetic and greatly esteemed guide and friend.

### A PROTEST AT MIDDLESBROUGH.

There has been a protest at Middlesbrough by a medical man against the small amount assigned by the managers of the Hospitals Saturday and Sunday Fund to the Nursing Institution. The total at the disposal of the Fund was £462, of which £221 was allocated to the North Riding Infirmary, £216 to the North Ormesby Infirmary, and £26 to the Nursing Institution. At the annual meeting Dr. Hedley said that the claims of the latter had been most inadequately recognised, and complained that a motion of his in favour of increasing the proportion to the Nursing Institution had not been considered before the division of the amount was confirmed. We think that the total sum is not at all worthy of Middlesbrough; but if, as Dr. Hedley affirms, the working men of the town are of opinion that the Nursing Institution should have the lion's share of it, the remedy is easy. They can support the Nursing Institution directly. It must be remembered that the Saturday and Sunday Funds were originally intended only for the benefit of the hospitals.

### FEVER HOSPITALS AND THE BIBLE.

We notice that a former patient in a London fever hospital writes to the daily press to complain that in his ward, with fourteen beds, there was only one Bible, "a very old musty-smelling one, with extremely small print." It may be urged that patients ought to provide their own Bibles, but if they did so they would not be allowed to take them away from fever hospitals on their discharge. This is a matter in which the sister might make a representation to the matron. If the authorities of institutions for the reception of people suffering from infectious diseases do not consider that they are justified in the purchase of Bibles—not of the smallest print—for the use of the sick, we are sure that an appeal in some of the papers would have the desired effect.

### ADMIRABLE PROGRESS AT DARLASTON.

An encouraging feature of the report of the Darlaston District Nursing Institute, which has recently been issued, is that the subscriptions of the working men amounted to £162, an increase of £12 on the previous year. The general subscriptions and donations, on the other hand, show a decrease of about the same amount. But owing to a special gift of £100, and to the sum brought forward, the credit balance on the account was £645. The Institute will now have £600 invested on mortgage of the rates of the city of Birmingham at 3½ per cent., and the committee hope that within a few years the income from the working men's contributions and the investments will suffice to maintain the organisation.

### RAISING THE WIND AT CREDITON.

The sale of work held at Newcombe, Crediton, resulted in about £80 being raised on behalf of Crediton Sick Nursing Association, which, after twenty-one years' useful work, found itself in need of funds to restore it to a position of solvency. The sale was opened by the President, Lady Audrey Buller, who alluded to the harmony which characterised the work of the Committee, the officers, and the nurses. She stated that, in addition to nursing, the Association provides necessaries for the sick, and sends patients to various hospitals and convalescent homes; it also employs a maternity nurse. There is an emergency fund, but the Committee are naturally anxious that it should not be used for ordinary expenditure. Sir Redvers Buller, in acknowledging a vote of thanks to his wife, said that when an association had existed for more than twenty years people were apt to forget the state of affairs which prevailed before it existed. This is often the case, but it remains the duty of the Committee of the Crediton Association to see that its claims to the support of the public, by means of regular subscriptions, are recognised.

### SHORT ITEMS.

The wedding took place on September 4, at St. Albans Church, Teddington, of Miss Adah Charlotte Stroughill, matron of the Victoria Hospital, Kingston-on-Thames, Surrey, to Mr. John Farmer Collinson.

### The Nursing Outlook.

"From magnanimity, all fears above;
From nobler recompense, above applause,
Which owes to man's short outlook all its charm."

### NURSES' LEAGUES.

There can be no doubt that every one is benefited throughout life by keeping in touch with the institutions which have most influenced their characters in early life. School and college friendships are amongst the most precious and lasting of all. We welcome, therefore, the movement to establish a nurses' league in connection with every hospital and infirmary throughout the country. The objects of such a league should include that of strengthening a feeling of unity between past and present members of the nurse-training school and staff, the publication of an annual report or journal, the holding of meetings at intervals for the discussion of topics calculated to interest the members, and the inculcation of a knowledge of the nursing movement throughout the country, its trend and meaning, so that each nurse may have her mind enlarged sufficiently to enable her to contribute her mite to the increase of the general usefulness and best interests of the nursing profession. Another useful purpose fulfilled by a league should be to keep a register of all the past and present members of the nursing staff of each institution, to keep in touch with those members, to record their advancement and promotion, and so to give each member strength for her work after she may long have ceased to be connected with the hospital where she was trained.

It may be well to give brief suggestions as to how to start a nurses' league in connection with a hospital or infirmary. The first step is for the matron, in conference with the superintendent or secretary, to hold a meeting of the sisters, nurses, and probationers at present on the existing staff. The matron will no doubt obtain from the superintendent or secretary valuable hints as to how to organise the business of such a meeting, and the general lines for business purposes which should be followed in regard to future meetings. The rules should make every old and worthy member of the staff eligible to join the league. Subsequent members should be required to hold a three years' certificate from the institution where they were trained, or hold such a certificate from some recognised training school and have been engaged on the staff of the league hospital for not less than a year. The honorary officers usually include a president, a treasurer, and a secretary; the matron for the time being is usually the president; the secretary is usually the sister who has shown the most aptitude for the duties of such an office; and the treasurer should be some one with a knowledge of business who will take care of the funds and economically administer them in the best interests of the members. The officers are usually elected for from one to three years; experience shows that it is well that every officer should come up each year for re-election by the general meeting. It is useful, too, that each president on retiring from office shall be added to the list of honorary presidents of the league. The committee should consist of from six to ten members, three of whom should retire annually and be eligible for re-election. The quorum for the committee meetings should be one-third of the total number of its members, and it should have power to make by-laws and regulations, subject to the confirmation of a general meeting. The members of the council ( ? committee) should be equally divided between resident and non-resident members of the league. The annual subscription of members, including the cost of the annual report or journal, should be from 5s. to 3s. 6d. per annum. Subscribers whose subscriptions are in arrear should forfeit the right to vote at the annual meeting. The date of the annual meeting should be fixed at a time of the year most convenient to the majority of the members, and all details connected therewith should be left to the president and committee. In addition to the annual meeting there should be sectional meetings during the autumn and winter, and a social meeting of all the members at least biennially. The journal of the league should prove a most useful and important document in the best interests of the hospital or infirmary, as it should record the promotion, advancement, and career of each nurse who has worked in the institution, together with her experiences, and an account of any interesting travel notes which she may be able to supply for publication in its columns. The more the members exert themselves to supply copy for the journal the more interesting will each of these publications become. It is essential for the success of each league that the annual meetings shall be held at the institution, for there everybody will be most likely to inhale the atmosphere of alma mater, and the first object of the league must be to promote its interests and to keep in touch with its progress from year to year.

It is essential, however, to the success of each nurses' league that it shall maintain its independence by keeping free from the influence of all individuals who may wish to capture the league for personal or political purposes. We have not space to go into this aspect of the question to-day, and must therefore defer it. It is necessary, however, to put all nurses upon their guard, for otherwise they may find themselves committed to opinions, and claimed as the supporters of organisations, for which they have neither sympathy nor respect. To succeed to the full a nurses' league must be so organised as to secure, as the first and main result of its work, the uniting together of all the workers who may have passed through each school for their mutual advantage, education, success, and happiness. Once introduce politics or petty personal intrigues, and the high promise of good which these leagues offer may be destroyed.

### The Care and Nursing of the Insane.

By PERCY J. BAILY, M.B., C.M.Edin., Medical Superintendent of Hanwell Asylum.

### I.—ANATOMY AND PHYSIOLOGY.

(*Continued from page* 314.)

*The Functions of the Nervous System* are very numerous and complicated, but it is only necessary here to give a brief and simple description of the general principles upon which it works.

I. The white matter, which we now know consists of nerve fibres, is the connecting link between various nerve centres or between a nerve centre and some other tissue or organ. Its function is always the same—namely, the conveyance of an impulse or message either from one nerve centre to another, or from one or more centres to some other tissue or organ, such as a muscle, or, again, from some other tissue, such as the skin or retina to a nerve centre. The nerve fibres in our body, then, wherever they may be found—that is to say, whether they form part of the white matter of the brain or of the spinal cord, or whether they form part of an ordinary spinal or cranial nerve, are like the telegraph or telephone wires that pass from one town to another all over the country, only there is this great difference, that, whereas in the case of any particular telegraph wire the message may pass in both directions along the wire, either to or fro, this is not the case with a nerve fibre, for any individual nerve fibre conveys its message always in one and the same direction, so that if a message is to be sent to any nerve centre, and an answer received back—a condition of things which, as we shall see later, constantly occurs in the everyday work of our bodies, two separate nerve fibres are necessary—along one of these fibres the message travels in the direction towards the nerve centre, and as such a message when it reaches the centre results generally in some sort of sensation, we may call such a nerve fibre a sensory nerve fibre (afferent). Along the other fibre the answer is returned, the message travelling in the direction away from the nerve centre. Now the commonest, or perhaps we should say the most readily recognisable, result of a message travelling in this direction is a muscular contraction which gives rise to some form of movement; hence we may speak of nerve fibres which convey messages away from a nerve centre as motor nerve fibres* (efferent). These two kinds of nerve fibres, afferent and efferent, are usually combined with one another in all the cranial and spinal nerves, and when this is the case the nerve is spoken of as a mixed nerve.

We have seen that each of the spinal nerves arises from the spinal cord by two roots, which almost as soon as they leave the cord unite with one another. These two roots are composed each of a particular kind of fibre, the anterior or front one being made up of all motor fibres, while the posterior or back one is made up entirely of sensory fibres.

II. The functions of the grey matter are of a much higher order, and are much less easy to understand than those of the white matter. It is in the cells of the grey matter that nerve impulses arise, or are received and interpreted into sensations, and the peculiar and special function of grey matter depends upon the vital activity of the nerve cells which it contains.

We have compared the function of the white matter, or nerve fibres, to that of the telegraph wires which convey messages from one part of the country to another. If we might continue our comparison, and apply it to the nerve cells of the grey matter we should say that these resemble the cells of an electric battery, which génerate the electricity which is the force whereby the telegraphic message is conveyed along the wire. But the results of the activity of nerve cells are very numerous and varied. Whenever a nerve cell passes from a state of rest into one of activity, such change of condition may produce one of either of the three following results:

1. Some mental or intellectual process, thought, etc.

2. The generation of a nervous impulse which passes out from the cell through, we will say, a motor nerve, producing some kind of motion.

3. Some sort of sensation—in this case the activity of the cell being awakened by its receiving a nervous impulse arising elsewhere and conveyed to it through a sensory nerve.

During the whole period of life there is a constant flow of nervous impulses outwards from the grey matter to the various organs and tissues of the body and in the contrary directions inwards to the grey matter from the various organs and tissues of the body. The former of these impulses (those travelling away from the grey matter) pass through what are called the efferent (carrying away from) nerves. For the sake of simplicity when dealing with the functions of the white matter, only one class of these nerves—the motor nerves—was mentioned, but it may be easily understood that there are many other results of those efferent or passing away impulses besides muscular contraction. For instance, such impulses control the secretion of the innumerable glands of the body, the tears, the sweat, the urine, the gastric, and intestinal glands, etc.; others, again, control the process of assimilation and the nutrition of the tissues.

The other set of impulses (those travelling towards the grey matter) pass through what are called the afferent (carrying towards) nerves. When these impulses reach the grey matter the result of the activity of the nerve cells which they thus awaken is generally some sort of sensation such as sight, hearing, taste, smell, touch, heat, pain, etc.

Having now obtained a general idea of the uses of the two kinds of nerve tissue, we may pass on to consider the special functions of the various anatomical divisions of the nervous system.

1. *The Functions of the Great Brain.*—We have seen that the grey matter of the great brain is arranged in what we may call two systems, the

* It should be mentioned here that the motor nerves form only one of several classes of efferent nerve fibres, as will be more easily understood presently.

outer crust or rind which we call the grey matter of the convolutions and the secondary patches which are more deeply situated and which are known as the basal ganglia. Now it is the former of these two systems which specially concerns us here, and we may leave the functions of the basal ganglia out of consideration altogether, for they are not yet very clearly understood and any attempt to explain them would lead us into realms which we have no need to explore.

In the grey matter of the cerebral convolutions resides the power of thought and that of originating motion, and here it is that the impressions which travel through the afferent nerves from the skin, from the muscles and joints, from the organs of special sense, etc., are received and interpreted into what we call sensations, awakening within us a knowledge of external events and of the condition of our own organs and tissues, the sum of these sensations being what we call consciousness. Hence we say that the functions of the great brain (cerebral convolutions) are briefly that:—

1. *It is the Seat of the Mind,* meaning thereby consciousness, the power of thought and judgment, memory, the exercise of the will, the emotions, etc. Of these functions we have nothing more to say in this place.

2. *It is the Seat of Motion and Sensation.*—Every voluntary movement we make depends upon and is brought about by muscular contraction, but our muscles have no power of themselves of passing from a state of rest into one of contraction; this can only be brought about by the receipt by the muscle of a nervous impulse or message. Every such nervous impulse or message arises in the grey matter of the convolutions and is conveyed therefrom to the muscles by a motor nerve-fibre. If at any time we change our condition from one of rest to one of motion, if, for example, when sitting down we suddenly conceive the idea that we will rise and walk across the room, the muscles which accomplish this movement act only in obedience to directions or commands received from the grey matter of certain portions of the convolutions. Such directions or commands or nervous impulses are the result of some sort of chemical activity in the nerve cells, of the nature of which we are quite ignorant. This chemical activity may be started by some stimulus received from outside the brain; for instance, in the example we have already taken, the movement may be induced because we feel a draught of cold air which gives rise to the thought that it would be wise to close a window or a door, and we rise to effect this purpose; or the thought or idea which results from such chemical action may arise entirely within the mind without any obvious external cause.

(*To be continued.*)

### The Nurses' Clinic.

### INJURIES OF THE LOWER LIMBS.

The injuries most frequent and common are those of the limbs, the majority are not serious enough to endanger life, but those of the lower limbs may enforce many days or weeks in bed and therefore the patient will need a nurse's care and attention.

Taking those of the lower limbs first, the injuries may fall under any of the following heads: (1) bruises; (2) hæmatomata; (3) wounds; (4) fractures—(*a*) simple, (*b*) compound; (5) dislocation of the ankle, knee, or hip joint. Bruises may cause a great deal of effusion and consequent swelling and pain; the patient should rest the limb raised on a firm pillow. An ice-bag or evaporating lotion or a lead and opium fomentation may be ordered.

Massage is soothing and beneficial, and by means of this the effusion and subsequent discoloration may be relieved and the work of the lymphatics and blood-vessels accelerated. A hæmatoma subsides usually with rest and application of cooling agents, such as those mentioned for bruises. Wounds will be treated with the usual antiseptic precautions. The deeper and the nearer large arteries or veins, the more care must be taken to prevent sepsis or puncture of the vessels near. Every nurse should make it her business to know where the large blood-vessels lie, so that she may have some idea how to arrest violent hæmorrhage in cases of emergency; she should also learn how and where to put on a tourniquet.

Fractures must be treated with care; by the time, however that the patient comes under the care of the nurse he has probably had the limb seen to and attached to a splint, so that the chief thing for the nurse to do is to see that the splint keeps its original position; if the bandages or straps and buckles slip, it must be reported at once, so that they may be replaced, otherwise there is the risk of movement and displacement of the fracture.

A simple fracture in good position should do well and allow the patient to start using the leg by degrees in about three or four weeks, unless it is a fracture of the femur, in which case the patient will have to lie in bed from six to eight weeks. If the patient is old or in an unhealthy condition frequently fractures will not unite without the assistance of wiring the ends of the bones together; thus held in apposition there is a much better chance of speedy and good union.

In cases where the patient is thin and old and there is every prospect of a lengthy stay in bed, it will be advisable to put him on a whole water-bed; the surface will be even, pressure will be avoided and consequently the risk of a sore back.

The compound fracture will be more difficult to deal with owing to the necessary disturbance to dress the wound, but if possible the limb is left on the splint during the dressing, an opening having been left when the bandages were applied.

Severe injuries, such as laceration or complete crushing of the foot or leg, will nearly always necessitate amputation. The chief dangers to cope with in these cases are: (1) hæmorrhage before operation; (2) shock and collapse; (3) secondary hæmorrhage.

The patient must be kept extremely quiet, encouraged to take plenty of hot fluids, and the limb must be well raised. This treatment applies to both before and after operation. The varieties of injuries, and especially fractures, will give the nurse a splendid chance of making the acquaintance of the many splints used for support, and she will do well to observe and ask questions as to kinds of splints and when and how to apply them. For a "Pott's" fracture a back splint and two "Cline's" or side splints will be used as a temporary support, and after the swelling has gone down

**THE NURSES' CLINIC**—*Continued.*

probably a plaster splint will be applied. The "Pott's" fracture will prove a more lengthy thing than an ordinary fracture of both bones higher up the leg, because some of the surrounding structures composing the ankle-joint are involved, and these tendons, ligaments, and muscles must be allowed to regain their normal tone and strength before the patient is allowed to use the foot to any extent. The dangers of getting about too soon may cause flat foot and much pain caused by pressure on the plantar nerves.

A simple fracture of fibula or both tibia and fibula will have to rest in bed until the swelling goes down and a plaster can be applied, after which crutches will be allowed, probably, for a little time each day. Some surgeons maintain that the patient is better to move about so as to improve the circulation and thus afford a better blood supply to the affected limb. Fractures of the patella will always require complete rest and immobility of the joint—a back splint or ordinary straight splint may be used. After the effusion has dispersed, the fragments may be wired together and union should take place and the joint permit of gentle movement in two or three weeks. Massage and gentle passive movements are often ordered by the surgeon to assist in regaining the normal amount of strength and movement.

Fractures of the femur are treated according to their position and the age of the patient. If the patient is a child there are various methods of treatment. For a fracture anywhere in the shaft of the bone the method may be used of slinging up both legs vertically, and by means of strapping, cords, and a pulley, attaching weights to the injured leg, thus causing enough extension to prevent overlapping of the ends of the bone and keeping them well in apposition.

A double "Bryant" splint proves a very comfortable and good way of treating femur fractures near the neck of the bone; here again extension can be applied by an elastic accumulator, or by weights hanging over the end of the bed.

A "Liston" splint is sometimes used all through, but the tendency it has to slip is dangerous and children are never to be depended upon to keep absolutely still, so that the other two methods will more often be used. Finally, a single "Thomas" may be worn in the daytime and the patient allowed to try crutches.

The "Hodgen" splint is popular in some of our large hospitals for treating adult fractures of the femur. Although the leg slung up may appear uncomfortable, patients frequently prefer this method after a "Liston" or back splint and extension. With the "Hodgen" splint the patient can lift himself slightly without injury to the fracture, and his own weight acts as extension against the opposition of the cords attached to the apparatus at the foot of the bed.

Patients suffering from any of these fractures are apt to get a sore heel from pressure of the splint or the bandage or strapping. The nurse must always report this, or if she is authorised to do so remove the bandages sufficiently to place a pad under the ankle, which slightly raises the heel and relieves the pressure.

Dislocations may often be reduced at once by the surgeon, or in more severe and recurring cases operation may be resorted to and the trouble remedied by wiring.

In the less severe cases where reduction is effectual at once, the joint must be rested for a few days and supported by a firm bandage. The surgeon may order massage to relieve accompanying effusion and swelling.

### Incidents in a Nurse's Life.

### A MYSTERIOUS AFFAIR.

My friend and I were private nursing on our own account in Dublin. Times were bad and work scarce, so I was very pleased when our former matron rang me up and said that she wanted a nurse for an urgent case, fifty miles "below" Galway. I hastily packed my basket, and caught the 7.15 from Broadstone, wondering much what the urgent case would turn out to be, as matron had supplied no particulars by wire. It was the 8th of December, and the night was intensely cold. Snow had fallen heavily some days before, and was frozen hard. I admired the beautiful night from my third-class window, shivering with cold, and was glad of the old rug which my landlady had pressed on me at the last moment.

The train arrived in Galway, "the City of the Tribes," at 12 A.M., and I alighted—the only passenger. A sleepy porter basket, and murmuring something about "the hotel," led the way. I followed, mentioning my destination.

"Glory be! ye are going there to-night?" quoth my guide. "Take me advice, stay here to-night, and go on first car to-morrow."

I, feeling as if nothing would stop me, objected. I also objected to the "hotel," as I should have to sit in the bar. There was nowhere else but the post office. I went there, and sat on one of His Majesty's mail-bags whilst the letters were being sorted. At last the mails were ready, and the "Bianconi," or elongated side-car, was ready, the driver perched up high in front. Again I was the only passenger. I looked at it ruefully, and felt very small, and, Irishwoman though I am, I confess to feeling very uncertain as to whether I could hold on to that great car.

With a cheerful "God speed ye, and mind ye don't lose the lady!" we were off. Indeed, I felt like being lost. I don't know how I stuck on. The cold was intense. My hands were numb, and the cold seemed to have seized on me. Nothing but the vision of the serious case I was hurrying to buoyed me up. The driver now and again looked round and shouted, "Are ye there, miss? Are ye frozen entirely? Faix, he must be bad entirely to have ye vinture such a night!"

The country looked lovely under the stars. Is there anywhere on earth like Connemara? The road wound round and round the mountain, and fresh charms appeared at every turn; and when the day dawned the lovely purple lights which hung over the hills, with mists rising from the lakes, only divided from the roadway by a low stone wall, would have delighted an artist.

At Recess at 4 A.M. it began to rain—cold, straight rain. I really was tempted to stay there until a later car, but felt it would be cowardly, so climbed another "long car," and finished the journey at eight o'clock, wet and cold and hungry. We drew up at a public-house—the house of my patient. I thought everyone very smiling for a house of sickness. All the anxiety seemed to be to get me comfortable.

After a change and wash I was taken to the patient. I found her sitting up in bed, eating a hearty breakfast. She greeted me kindly, adding, "Pa lost his bet, nurse. He bet that no one would send a nurse out to drive across Connemara such a night as last night, and here you are!"

I was naturally rather indignant, but found that "pa" was not often sober, and was at heart a kindly man, and that it was the person to whom the bet was made who wired—and wired so efficaciously that he won his wager. I stayed with my patient for the week, and learned to like the people, finding plenty to do in one way or another. Finally, the patient travelled back to Dublin with me in daylight and by easy stages—a very different mode of travelling to my first experience of Connemara by night.

### A Week's Experience in a Canadian Hospital.

At the close of a four months' post-graduate course in district nursing in one of the principal cities in the Dominion, I received orders, rather unexpectedly, to proceed to a little hospital in a small town situated on one of Canada's inland seas. The journey could be taken either by land or water, and, having no deep love for the latter, especially in its stormy moods, I proceeded by train. As we pursued our journey the towns grew smaller, and the scenery became more wild; bush and swamp, and streams filled with floating logs met the eye everyhere. After travelling a day and a half I reached my destination, at least as far as the train was concerned. Tired and train-sick, I got out of the compartment and looked about me. I could only see one house in what looked like miles of bush and small trees, and I began to wonder what on earth they wanted with a hospital at all. Upon inquiring of the station-master, I was informed that the town was three miles away, and that I should have to take the bus to get there. Eventually I found a solitary vehicle, rather unlike our English busses, standing at the back of the station, and in it I went on the rest of my journey. The roads were fearful. I expected every minute to be shot over the wheel. In about three-quarters of an hour we reached a fair-sized village, crossed a river filled with floating logs, and found ourselves at the hospital gate. I was agreeably surprised to discover that though the hospital was small, it was quite up-to-date. There were four private rooms, containing one bed each, very well furnished, and with polished floors. There were also two public wards, one for the women and the other for the men. The women's ward held three beds, and the men's ward four. The hospital was three-storied, with a bath-room on either floor. The theatre was well supplied with most of the necessary accessories. There was only one other nurse besides myself, and I received a warm welcome from her. English nurses, by the bye, do not always receive a warm welcome from their Canadian sisters. I retired early to rest to recover from the journey, and next morning started upon my duties. The first thing to do was to get the wards straight. I learned that I was expected to sweep and dust them all, the top landing, and the bath-rooms, and to keep the baths clean. The maid attended to the front hall and mopped the wards once a week, but otherwise never came near them. I felt very much like turning up my nose. After three years' general training and one year of midwifery one does not like returning to what is considered probationers' work. Moreover, in the hospital where I trained we were not expected to do more than dust the wards. However, things are different in the back woods of Canada; the Canadian servant is a very particular person, and about as hard and expensive to get as rare china. In due course of time I found that every two alternate nights I had to do night duty, and as the opportunity for sleep in the day-time was scant, one was practically on duty 18 hours out of the 24 every two alternate days. For the first day we had only two patients—one with typhoid fever; the other a stomach case. On the third day a patient came in for operation—perinorraphy, ventral suspension, and appendectomy. On the morning of the operation I was asked to give the chloroform, but declined, as I was not used to giving chloroform; so another medical man had to be called in. There were only two doctors in the whole town, and not another within a radius of 40 miles. My fellow-nurse assisted the doctor, a lady of the town, not a trained nurse, attended to the sponges, and I did the handing. The next two days passed quietly enough, except for a small operation and plenty of other work. The nurse in charge had been away for holidays, and things had got to sixes and sevens. On the morning of the sixth day the nurse in charge went off for the day, leaving me to my own devices. She had scarcely got safely away when an emergency case came in—a maternity case, with shoulder presenting. The patient had been ill several days; she had not called in the doctor, and consequently was in a very exhausted condition. It was found impossible to deliver her, so an operation had to be resorted to, and cesarean section was performed. There was only the doctor and myself, therefore I had not only to give the chloroform, but had also to help as much as possible. All the dressings and sponges had been sterilised in small packages, so that it was possible to handle them without touching the contents. The patient lived till the evening of the next day, and then died from shock.

In the meantime three other patients came in, and finally I ended my first week by assisting the doctor in a post-mortem examination. So at least I could not complain of monotony.

### A Floral Fancy.

A musical murmur, audible only to listening fairies, came from the table at the top of the hospital corridor, on which were placed the vases of flowers brought at night time from the adjacent wards. There were many of those vases there to-night, the result of a flower service held in the afternoon at a neighbouring church.

The sound was hushed as a night nurse, her pale uniform dimly outlined in the gloom, came swiftly down the corridor, passing so close to the table that her gown touched an overhanging spray of lilac. When she had disappeared through a heavy swing-door, the faint, silvery murmur arose again, and was thus interpreted by an inquisitive fairy hovering near to listen to what the flowers might say.

"Now, my children," said a crimson rhododendron in grave, paternal tones, "let us recount the way in which we have justified the opinion which that clergyman evidently held of us when he told the children in church this afternoon that we should act as little rays of sunshine in the hospital wards. Since to us is given the power of reading the thoughts of human beings, a few of us may have something of interest to tell."

There was a shy rustling amongst the flowers, each urging her neighbour to speak. Then from a bunch of delicate white narcissi one whispered timidly :—

"My tale is quickly told. The sister placed the vase in which I stand before a collier who had been brought into the ward the day before, severely injured. He was in great pain, but he drew me towards him, and as he breathed my fragrance I carried to him thoughts of the beauty of the earth, of God's goodness and of the purposes which He accomplishes by strange means. Before I left the ward many of the lines of suffering on his face were smoothed away, and I heard him ask that I might be placed beside him again in the morning."

A delicate clump of forget-me-nots spoke next.

"I was taken to a women's ward. 'Are not these pretty?' said a nurse, pointing to me. At her words a girl lying in a corner bed—a girl with a white face and

A FLORAL FANCY—*continued.*

troubled eyes—turned and looked earnestly at me, and I saw the tears commence to fall. Mischief-makers had recently made her question the loyalty of her lover. I reminded her of a day spent with him in the country, a day full of pure and tender memories, when he had given her some of my flowers in token of fealty. I think the remembrance made trust triumph over doubting, for in the evening she wrote a letter which brought peace to her heart."

The forget-me-not's friends smiled approvingly, for all flowers are keenly interested in the doings of lovers. Then the snap-dragon gave a little tinkling laugh.

"My travels took me into a children's ward. There was a very cross baby there—brought in that morning and suffering from acute home-sickness. How he did yell! The tired-looking probationer in charge of him looked at her wits' end, but, catching sight of me, a happy inspiration seized her. She lifted me out of the vase and snapped my poor jaws backwards and forwards until I thought my head would tumble off. But didn't that baby like it! The tears vanished, all the cross wrinkles died away, and a dimpling smile appeared, which deepened presently into a chuckle of delight. I can't help laughing myself at the memory of it."

The other flowers beamed sympathetically. Then a sweet-william gave a little sigh.

"I was placed on a locker beside a man dying from the effects of an evil life. He looked at me for many minutes, and I saw the shadow of a painful memory in his eyes. Then he hid his face in his hands, and I knew that the tears were falling. He had seen a picture—a thatched cottage in the midst of fields ripening to the harvest, a tiny garden in front holding a wealth of sweet-williams—the favourite flower of the mistress of the little household; she herself standing on the threshold lovingly watching a boy radiant in innocent joy, as, manfully handling a very large spade, he essayed a task of gardening. Fifty years ago he had been that child—and he had broken that mother's heart."

There was a sorrowful silence. Then—

"I am afraid I have not done much," whispered a purple pansy. "A little seamstress, her illness caused by hard work and close confinement in a stuffy alley, laid my petals against her thin, white face. She thought of sweet country air, of sunny, old-fashioned gardens and wide, green spaces, and smiled."

"When I was carried into the ward," said a big peony with frank conceit, "I was placed, on account of my imposing size, in a prominent position on a centre table. On my entrance a political dispute of great bitterness was raging between two crusty old men, but on catching sight of me their voices ceased, to be raised a moment later in joint admiration of my size and magnificence, and an amicable discussion followed as to the conditions best suited to my development."

"A nurse kissed me as she placed me in water," said a sprig of mignonette. "A passing friend teased her for this—as she termed it—display of sentimentality, but she laughed and said, 'I can't help it. It does remind me so of the garden at home. Just fancy! Only a week now to my holidays!'"

Then a lily-of-the-valley spoke—softly, very softly.

"A sister carried some of my flowers to a screened-off corner where a little dead child was lying, the heart-broken mother weeping bitterly at the bedside. She placed them in the tiny hands, and the woman lifted her face and tried to sob out a word of gratitude. The small act of tenderness seemed to ease her sorrow for a moment, for it taught her that although her little one had died amongst strangers, they were strangers with kindly, compassionate hearts."

Hush-sh-sh!

A nurse had entered the corridor, and suddenly the solitary electric light was extinguished. Through the long windows the delicate light of approaching morn showed itself. In the distance a bell clanged noisily; the hospital was awakening to another day of busy routine. The timid flowers' conference closed abruptly, and the listening fairy stole away.

### Appointments.

Abingdon Cottage Hospital.—Miss Jane Claudine Witchell has been appointed matron. She was trained at the London Hospital, and has since been sister at the Sussex County Hospital, Brighton, and staff sister at Queen Charlotte's Hospital, London.

City Hospital, Lodge Moor, Sheffield.—Miss Florence E. Carter has been appointed night superintendent. She was trained at Fir Vale Infirmary, Sheffield, and the South-Eastern Hospital, London. Since then she has been charge nurse at the Wanstead Cottage Hospital, Essex; charge nurse at the Eastern Hospital, Homerton, London; and the Small-pox Ships, Long Reach, Dartford; and ward sister at the City Hospital, Sheffield. She was also engaged in private nursing for the Brooklyn Institute, Anerley. She holds the certificate of the Central Midwives Board.

Melbourne District Nursing Society.—Mrs. Hawkes has been appointed sister-in-charge. She was trained at the Alfred Hospital and the Women's Hospital, Melbourne, and has since been chiefly engaged in private nursing.

Potter's Bar Cottage Hospital and Dispensary.—Miss Harriett Griffith has been appointed matron. She was trained at the Stockport Infirmary, where she has been theatre staff nurse. She has since been sister at the Isolation Hospital, Coventry; sister of men's ward and night sister at Stockport Infirmary.

North Staffordshire Infirmary, Hartshill.—Miss Amy Howell and Miss Ada Mitchell have been appointed staff nurses. Miss Howell was trained at Oldham General Infirmary, and Miss Mitchell at the Royal Southern Hospital, Liverpool.

Royal Berks Hospital, Reading.—Miss Edith A. Addison has been appointed office sister. She was trained at Westminster Hospital, and has since been ward sister at West Ham Infirmary, Whipps Cross Road, and superintendent of night nurses at the City Hospital for Infectious Diseases, Walker Gate, Newcastle-on-Tyne.

Ruchill Hospital, Glasgow.—Miss H. G. Landles has been appointed matron. She was trained at the Western Infirmary, Glasgow, where she has also been sister of the outdoor and electrical departments, surgical ward sister, and superintendent of night nurses. She has since been matron of Ochil Hills Sanatorium, Kinross, and matron of Knightswood Hospital, Partick.

### Wants and Workers.

Will anyone lend a wicker bath-chair for use of sick people in a country parish? Must be in good condition as the district is hilly.—Miss E. Titherington, Bramshott Rectory, Liphook, Hants.

### Everybody's Opinion.

[Correspondence on all subjects is invited, but we cannot in any way be responsible for the opinions expressed by our correspondents. No communication can be entertained if the name and address of the correspondent are not given as a guarantee of good faith, but not necessarily for publication. All correspondents should write on one side of the paper only.]

### LENDING UNIFORMS.

"Winsis" writes: In a certain town a fair is held yearly, and the nurses there lend their uniforms to ladies to collect money for their hospital. Surely this is not fair to our nursing profession, as the uniform is a part of a nurse, and, I consider, should be kept sacred and only worn by her. Can we wonder why our uniform is held with so little regard when nurses are so indiscreet?

### SECTARIANISM IN LONDON HOSPITALS.

The Matron of the Stewart Intitution, Chapelizod, writes: In reply to "Medicus" *re* "Sectarianism in London Hospitals," I would like to state that during my matronship of a large provincial hospital a Roman Catholic candidate was accepted for training who subsequently was appointed by my successor as a sister of a male medical ward. Also in my training school (the London Hospital) "Medicus" will find that religion does not debar a nurse from promotion.

### THE COTTAGE HOSPITAL MATRON.

"Reg" writes: It is very true what "A Matron" says in the article headed "Cottage Hospitals and some of their Difficulties," but there is another side to be heard. In certain hospitals, which, during some months of the year, have only one or two patients, how can the full staff occupy their time while waiting for more patients? Most cottage hospitals are kept up by subscriptions, and it would fall heavy on the funds to keep such a staff simply waiting in the slack months; and a good many small cottage hospitals have several such months, during which there are only one or two patients to look after. It is very difficult to decide what should be an adequate staff, because, on the other hand, for a few months a staff similar to that mentioned in the article would be only just sufficient, and without it the poor matron and the said probationer would inevitably get overworked. I speak from experience, as I am a matron of a cottage hospital, and I wish that there was an easy solution of the difficulty. But I cannot ignore the other side.

### AMERICA AND ENGLAND.

"B. S. A." writes: I think that "American Nurse" should be the last to find fault with our doctors and nurses. We are told that charity begins at home, and "American Nurse" will find much scope to right her wrongs nearer home. I have recently returned from America after having some six months' private nursing there. During that time I met and nursed for about fifteen medical men, and, with one or two exceptions, I found them "lacking" in all that we look for in our own doctors—*i.e.* refinement, courtesy, and consideration for their nurses; in fact, these qualities were conspicuous by their absence. I cannot say that I was any more impressed by the American nurse. From my experience the almighty dollar plays a much more important part in her life than whether her patient will recover or not. Patience, self-sacrifice, and accommodating oneself to circumstances, which we are taught in our hospitals, are qualities which I found unknown to the American nurse. In fact her one prevailing thought seemed to be how long her case would last, and how many 21 dollars she would get, and would she be able to buy that duck of a hat and costume and go away for a week's holiday when she left her case? I also very soon realised the disloyalty which exists between American doctors and nurses. It is no uncommon occurrence for the medical men to inquire even of the servants, if they like the nurse, and I have known of one or two instances where the nurse was dismissed because the cook or some other maid did not like her. I also heard of two cases where nurses, not liking the doctor, prevailed upon their patients to change and have a doctor of their own choosing. No wonder that poor "American Nurse" found our doctors and nurses disappointing, for they naturally displayed qualities rarely met with in doctors and nurses of her own country.

### IGNORANT MIDWIVES STILL ABROAD.

"An Inspector of Midwives" writes: On reading your note, "Ignorant Midwives Still Abroad," the thought struck me that perhaps a few of my own experiences as inspector of midwives might do a little to show how almost impossible it is to insist on the rules of the Central Midwives Board being carried out, and how impracticable is the working of the Act in country districts unless one is to leave the poor mothers without any help at all at the most critical time in their lives. There are in my district some seventy or eighty midwives practising who are registered, and I suppose about twenty unregistered women who go out (or are supposed to do so) "only with the doctor or in emergency," although one woman I know goes out quite openly, secure in the knowledge that, as she has taken down her sign and calls herself "nurse," the law cannot touch her until 1910. Out of all those women there are only four who have received any training whatever, quite half are women between sixty and seventy-five years of age, and many of those can neither read nor write. The first outlying district I visited boasted one registered midwife, an old woman of seventy-four, whom I found lying in bed, suffering from an attack of "brownkitus," and bewailing the fact that she was getting past work. "Ah sall hev ter give oop; Ah canna do't no mooar; thou mun fin soomun else." Poor old soul! She could neither read nor write, and one could not help but feel how utterly absurd it would be to try and teach a woman of her years new methods. Yet, unless the State steps in, who is to take her place? There is not enough work to tempt a trained midwife to do so, and certainly not enough to enable her to live. The next one was such a cheery little body, spotlessly clean in house and person, with snow-white hair and such bright eyes. And how proud she was to exhibit her Re-gees-ter! "Ma son wretes it doon fer me. He be a rare gude scholard." It certainly was very well kept, although I was somewhat puzzled at the word "Right" under the heading of Presentation until I found that it was "right" in a double sense. The duration of first, second, and third stage I found quite beyond them all. Her bag and its contents—well, it showed that she was doing her best, and it also showed me how very necessary it was that I should have a bag, for I felt that one practical demonstration would do far more good than an hour's talking. This is a fair sample of all in the outlying districts; in no case where a register had been obtained was it kept by themselves. "Ah be no scholard" was the general cry. To conclude, I do not think I can do better than relate one incident where I found the lady of the house busily engaged in washing her little grandson, whose mother was "oop't mill," and who had had a "mishap oop't schule, the very fust toime he'd a doon sut a thing, fer he wor a right clane un, he wor"; and you may judge of my surprise when the husband (who had been sitting the far side of the table) got up to assist in the explanation, and revealed himself minus his trousers! He trotted about quite unconcernedly, telling me that "towd 'ooman wor gooin' ter mend ma britches, and her dos'na want me ter put ma black 'uns on." However, the gentleman had to put on his "black 'uns"; and when he proceeded to do so in front of me, as though it was quite the most correct thing to do, I felt that my control was fast leaving me. Fortunately he soon made his exit, and the little grandson, having by this time been reduced to a very tearful, if "clane," state, I was able to make my "inspection," which in this case consisted of the lady only, both register and bag being conspicuous by their absence.

### A Book and its Story.

### SPIRITUALISM IN FICTION.*

Mrs. Pugh's new novel touches heights and depths. It reaches from the serene heights of affection between Victor Levigne, a spiritualist, and his motherless daughter Amarant, to the less worthy plane where demonstrations of so-called "spiritism" are given in the drawing-room of a suburban widow lady, which lead, on one notable occasion, to a hasty break-up of the "seeaunce"—the parlourmaid's term for the meeting—in consequence of the unexpected presence of the Vicar of the parish, who appears in the flesh in reply to repeated calls from the widow through the darkness for some sign from the hostess's dear departed. The Vicar, since the arrival of Mr. Levigne and his daughter in his parish, has heard that they are spiritualists. Ridiculous rumours reach his ears. He had written to Mr. Levigne on the subject, but a polite but firm refusal to see him is the only reply that came in response. By degrees the extraordinary stories connected with the old, deserted mansion recently taken by Mr. Levigne, gain credence. "Rumours, at first vague, slowly became definite. Sounds were heard, sights seen, in the Tree House that could not be attributed to any human agency. For instance, there were moans at midnight in the corridors, so weird and appalling mortal man could not possibly have uttered them; bells rang where no bell had ever hung; balls of light rose up in dark rooms through solid chairs and tables. . . . Finally—this, it was understood, was what made the between-maid decamp—Mr. Levigne, lying on his bed, six foot six by four foot six in size, and solid brass and iron, was borne by invisible hands down through the floor of his bedroom and out through the shuttered window of the drawing-room to the garden. These stories naturally incensed the Vicar. It was inconceivable that such ridiculous nonsense could be circulated and believed in the nineteenth century, within twelve miles of London. But his annoyance was not wholly due to the credulity of his parishioners; it lay chiefly with Mr. Levigne, who was himself the unwilling cause of the existing state of affairs. He admitted that "towards that gentleman he felt as might have felt a priest in earlier times if a witch had been denounced before him."

The Vicar at once begins a crusade in the parish against the sinister influence supposed to be at work. His surprise visit to Mrs. Hughes was the outcome of a declaration repeated to him, which she had made in reference to Mr. Levigne. "She had the temerity to say before several people that she thought 'she was developing clairvoyance as well as clairaudience,' and she meant by hook or by crook to consult Mr. Levigne about it." At the outset, on his arrival at Mrs. Hughes's house, he is confronted with a difficulty. He has been kept waiting at the door; it is raining hard. When the bell is at last answered, he steps at once inside, and, shutting it himself against the wind and rain, takes off his wet overcoat. Mrs. Bevan is an old friend, and he claims a friend's privilege. But upon standing aside to allow the maid to pass and announce him, he is astonished by being told that her mistress is not at home. Through the open door of an adjoining room he sees signs of festivity in the remains of a dainty afternoon tea, and the sound of voices is distinguishable in the drawing-room. Among them that of Mrs. Hughes is clearly heard. Again the inquiry is made, "Did you say 'not at home'?" and this time there were volumes of sarcasm in his voice. The maid broke down. "She said it was to be 'Not at home,' sir, to anyone but those invited to the seeaunce." "To the what?" "The seeaunce, sir, the fortnightly seeaunce." The Vicar was shocked by the announcement. Not waiting to be announced, he mounts the stairs, "the light of battle shining in his eyes." The door of the room is ajar; he pushes it softly open and enters. In the dimly lit room he was not seen. When he is accustomed to the light he finds a dozen or so of young people, all parishioners. He is the unwilling spectator of a séance in which Mrs. Hughes is taking the lead. She is making a final appeal for a communication from the spirit world. "Three loud raps answered her. There was a stir of agitation. 'Have you come,' continued Mrs. Hughes, her voice shaking a little as though she were losing her nerve, 'from the place of spirits to guide, to comfort me?' There is a stir near the door. Something dark moves, advances a step; the sitters jump up and leave the tables. One of them turns on the gas, and, amidst the confusion, the Vicar is recognised. For a moment Dr. Bevan stood at one end of the room, the 'seeaunce,' its nerves completely unstrung, at the other." Mrs. Hughes excuses herself, in answer to the Vicar's protestations, by saying that the séance had taken place solely in order to protect her from scoffers. "Scoffers," cried the Vicar; "Mr. Lucas, who has left the room, is one of the worst of scoffers" (Mr. Lucas had turned on the light and fled at the sight of the Vicar). "I perceived at once that he was amusing himself, and making a laughing-stock of you all. I consider his conduct disrespectful in the extreme. . . . What matters—what grieves me—is that all of you, carefully brought up, carefully taught members of the Church, have this evening been guilty of what I pronounce to be profanation and impiety, if not an actual tampering with the devil. . . You are children playing with dangers you do not understand." Mrs. Hughes assures Dr. Bevan that nothing would frighten them more than a direct revelation of the powers of darkness. "What we sought," went on Mrs. Hughes falteringly, "was perfectly harmless—just a touch of a vanished hand, a sound of a voice that was still, a word from one gone before." "Rubbish!" said the Vicar, forgetting his manners in his indignation. "Your husband was a sensible man, Agnes, and he didn't talk in raps. Do you suppose that his immortal soul is reduced after death to such a state of imbecility that he will come to a tea party on a wet afternoon and rap on a table that is being jiggered about by a rude young man?" Dr. Bevan goes home and writes a stormy letter to Mr. Levigne, begging him to refute the rumours in circulation about the happenings at The Tree House, which he considers are responsible for the scene he has just witnessed, and begging, in order to allay suspicion, and prove the rumours to be false, that he will appear at church on Christmas Day with his daughter, promising, in return, if Mr. Levigne consents, to see him, and allow him to demonstrate the truth of his theories of the possibility of communion with the dead. Mr. Levigne regretted, in reply, that he "was unable to explain objectively in the course of an afternoon call, truths that were outside the region of mathematics. Neither could he attend church on Christmas Day with his daughter."

The character of Amarant Levigne is a singularly sweet one. The book is divided into three parts. The first treats of the home life of the father and daughter at Tree House. Readers interested in the subject of spiritualism will find this and the third part, perhaps, most interesting. The second part takes Amarant, after the sudden death of her father, to the South of France, where, amid people, and scenes divergent to previous experience, she meets with thrilling adventures, which deepen the sadness of life for her.

---

* *The Unguarded Taper.* By Helen P. Lewis (Mrs. J. G. Pugh.) John Long. 6s.

### Notes and Queries.

**REGULATIONS.**

**The Editor is always willing to answer in this column, without any fee, all reasonable questions, as soon as possible.**

**But the following rules must be carefully observed.**

1. **Every communication must be accompanied by the name and address of the writer.**
2. **The question must always bear upon nursing, directly or indirectly.**

**If an answer is required by letter a fee of half-a-crown must be enclosed with the note containing the inquiry.**

*Babies' Home.*

(264) Can you tell me the name of any hospitals entirely for babies?—*Baby Lover.*

There is an Infants Hospital in Denning Road, Hampstead, London, N.W.

*Stewardesses.*

(265) Which of the big liners carry trained nurses; also where should I apply for such a post?—*District Nurse.*

You can write to the Booth Steamship Company, Adelphi Terrace, Strand, W.C., or the head offices at Liverpool, though there are seldom any vacancies. Some other lines carry trained nurses as stewardesses. The life of a stewardess is most wearisome, and there is scarcely any relaxation.

*Children's Hospital.*

(266) Is it necessary to have general training as well as children's training for the post of staff nurse in a children's hospital?—*Sydney.*

No. Promotions are frequently made from nurses entirely trained in a children's hospital.

*Dismissal.*

(267) A nonsensical letter I and another nurse wrote to a young man came into the hands of the matron of our hospital. She reported to the Board, and we were at once dismissed. Could we not demand to appear before the Board, and put our conduct in the best light; and should we have been dismissed for our conduct?—*An Inquirer.*

It is impossible to advise you, not knowing all the circumstances; but if you feel that your action admits of extenuation, certainly appeal to the Board.

*Chiropody.*

(268) Where could I learn chiropody, and if, when learnt, could I obtain a living by it? Do any hospitals employ or train chiropodists?—*Parade.*

You must become the pupil of a good practitioner in chiropody, and through your teacher you might secure a connection. It is possible to gain a living by chiropody. The hospitals do not train, nor have they a chiropodist on their staff.

*Canada.*

(269) Can you give me the address of maternity hospitals in Canada?—*Newhall Street.*

Ottawa House of Mercy Lying-in Hospital and Maternity Hospital in same town; Montreal Maternity Hospital.

*Midwifery.*

(270) Can you tell me where I can get midwifery training free?—*Hockey.*

Write to the secretary of the Association for Promoting the Training and Supply of Midwives, Dacre House, Dean Farrar Street, Westminster, S.W.; the secretary, Rural Midwives Association, 47 Victoria Street, Westminster, S.W., or to the Beds Rural District Association, Lansdowne House, Beds.

*Mental.*

(271) Can you tell me of a home where a lady mentally afflicted could be received for £1 per week?—*Cadogan.*

You will find a list of private asylums in Burdett's "Hospitals and Charities" and in "Medical Homes," both from the Scientific Press, 28 Southampton Street, Strand. But your best plan would be to advertise.

*Preliminary Training.*

(272) I should be glad to know of a preliminary training establishment where I could study before entering a hospital.—*Lynton.*

We know of no such establishment, and what you propose is quite unnecessary if you join such hospitals as the London or Guy's, where all probationers undergo preliminary training before entering the wards.

*No Institution Training.*

(273) I have relieved midwives on their weekly leave. Would this experience be sufficient to gain the C.M.B. certificate?—*Perplexed.*

No, as you are not trained. You should have applied in the period of grace, though we doubt if you would have been successful then. Now you must train at a recognised school.

*Address Wanted.*

(274) Can you give me the address of a specialist for a friend?—*Mildura.*

We cannot do this, but a list of specialists will be found in "Medical Homes," the Scientific Press, 28 Southampton Street, Strand.

*Queen's Nurse.*

(275) Can you tell a nurse with three years' certificate where to apply concerning becoming a Queen's nurse?—*Spring.*

The Lady Superintendent, Queen Victoria's Jubilee Nursing Institute, Victoria Street, S.W.

*Children's Nurse.*

(276) Can you tell me where to apply as nurse in a children's hospital?—*Anxious.*

The Alexandra Hospital for Children with Hip Diseases, Queen Square, W.C.; the East London Hospital for Children, Shadwell, E.; and St. Mary's Hospital for Sick Children, Plaistow.

*Dispensing.*

(277) I should be obliged if you will tell me the cheapest way to train as a dispenser.—*Felixstowe.*

Write to the Pharmaceutical Society, 17 Bloomsbury Square, W.C.

*Cairo.*

(278) Can you tell me of one or two nursing homes in Cairo? *Anxious.*

Write to the Anglo-American or the Kasr-el-Ainy Hospitals in Cairo. If unable to give you employment, they might be able to give you useful information on the subject. Enclose a stamp for reply.

*Home for Nervous Person.*

(279) Can you tell me of any home where a young woman suffering from nerves could be received? A payment of 5s. weekly is possible.—*Somerset.*

Possibly the best place would be a convalescent home. There are numbers of these, but the charge is usually higher. A very full list is to be found in Burdett's "Hospitals and Charities," Scientific Press, 28 Southampton Street, Strand, W.C.

*Disabled Nurses.*

(280) Can you tell me where assistance for disabled nurses can be obtained?—*Stranded.*

The members of the Royal National Pension Fund for Nurses has a fund to assist members in distress, but unless you are a member we fear we cannot hold out any hope to you.

*Plague Nursing.*

(281) Can you give me the address of the Plague Nursing Association in London?—*St. Pancras.*

We do not know of such an organisation. If you mean for India, you had better write for information to the Under Secretary, India Office, St. James's Park, S.W.

*Neurasthenia.*

(282) Do you know of any free institution where a lady suffering from neurasthenia could be admitted for Weir-Mitchell treatment?—*Matron.*

Possibly some of the convalescent institutions may apply the Weir-Mitchell treatment, and, if so, perhaps our readers can help. There would certainly be a charge of 10s. weekly and upwards.

*C.M.B. and Partnership.*

(283) *Matron*, 15 Beverley Gardens, Barnes, S.W., writes that she would be glad to see *C.M.B.*, as she is setting up a small private home in the autumn.

*Co-operation.*

(284) Can you tell me the address of the principal nurses' co-operation in London?—*Aberdeen.*

The Nurses' Co-operation, 8 New Cavendish Street, W.

*Male Nurse.*

(285) How can I obtain free training as a male nurse?—*Portsmouth.*

There is no training school in general nursing for men. The National Hospital for the Paralysed and Epileptic, Queen Square, Bloomsbury, trains attendants, and the Army offers good opportunities for learning nursing in the Army hospitals.

**Handbooks for Nurses.**

Post Free.

"How to Become a Nurse: How and Where to Train." 2s. 4d.
"Nursing: its Theory and Practice." (Lewis.) ... 3s. 6d.
"Nurses' Pronouncing Dictionary of Medical Terms." 2s. 6d.
"Complete Handbook of Midwifery." (Watson.) ... 6s. 4d.
"Preparation for Operation in Private Houses." ... 0s. 6d.

Of all booksellers or of The Scientific Press, Limited, 28 & 29 Southampton Street, Strand, London, W.C.

### Notes from India.

CALCUTTA.

In Calcutta one is surprised to find that the hospitals, together with many schools and orphanages, are under the dominion of the Sisters of St. John the Baptist, Clewer.

There are the Presidency General, the Medical College, and the Eden. the other hospitals, such as the Campbell, being mostly for natives or infectious cases.

In the first three hospitals the staffs are principally Eurasian. The sisters, of course, untrained, go round with the doctors and take their orders. The food for the staff at the Presidency, though suitable for the Eurasians, who are quite content with constant curries and other Indian dishes, is trying for European nurses.

Attached to the Presidency Hospital, and where the sisters live, is the Canning Home, bequeathed as a memorial of the late Lady Canning, but, oh, how different it would look were it under the superintendence of a trained English matron! At present the rooms are shabby, neglected, and not too carefully looked after, although there are constant charitable performances, balls, etc., under viceregal patronage, being given for the support of the hospital and private nurses' institution—the only one in Calcutta—which is attached The larger proportion of the patients are, as in most colonies, of the paying order.

Private nursing in Calcutta is far from being a paying concern, five to seven rupees (a rupee is one shilling and fourpence) being the charge. Rent is very high, and the climate during the summer months almost unbearable. There is, however, a great paucity of well-trained European nurses in the town, probably owing to all these adverse conditions.

BOMBAY, DARJEELING, AND BURMAH.

St. George's Hospital, Bombay, has much improved under the new *régime*. Miss Mills has now for some years been matron, and the All Saints' sisters are a dream of the past. Eurasians are no longer admitted on the staff.

In Darjeeling there is the prominent sanitorium, also nursed by the sisters. There are several grades of patients, the Tommies being frequently sent up from Calcutta to convalesce. There are many Dufferin hospitals scattered over the country, of course for women's work. Some are styled Ray Dufferin, the native Rajahs of the districts subscribing in large measure to the fund. There is also a Dufferin Hospital in Calcutta. There are others in course of erection. Burmah is badly off for nurses. The hospital at Mandalay, which contains 150 beds, twenty being set aside for Europeans, is often short-handed. They have a matron and five European nurses. The climate of Burmah is very trying, and in Mandalay a short time ago there was such a terrific outbreak of plague that two-thirds of the inhabitants fled, and business was practically suspended.

GENERAL HINTS.

To any nurse coming out for the first time to India the want of knowledge of Hindustni is a great drawback, as everywhere, except in Madras Presidency and Ceylon, the servants and workpeople generally speak no English. Hence many good hospital appointments are given to locally-trained "nurses," although their certificates are known and acknowledged to be of little value. There is a hospital at present advertising for both lady doctor and matron, who must have a good working knowledge of Urdu, a far more difficult language than Hindustani. Otherwise Europeans in all occupations are very highly thought of, so few of those born in India itself being of pure race.

### For Reading to the Sick.

A THANKFUL HEART.

My heart gives thanks for yonder hill,
That makes this valley safe and still;
That shuts from sight my onward way
And sets a limit to my day;
That keeps my thoughts, so tired and weak,
From seeking what they should not seek.
On that fair bound across the west
My eyes find pasturage and rest,
And of its dewy stillness drink,
As do the stars upon its brink;
It shields me from the days to come,
And makes the present hour my home.

*Louisa Bushnell.*

"As our day, so is our strength. If the trials of many years were gathered into one, they would overwhelm us; therefore, in pity to our little strength, He sends first one, then another, then removes both and lays on a third heavier, perhaps, than either; but all is so wisely measured to our strength that the bruised reed is never broken. We do not enough look at our trials in this continuous and successive view. Each one is sent to teach us something, and, altogether, they have a lesson which is beyond the power of any to teach alone."—*H. E. Manning.*

"The deliverance of the soul from all useless and selfish and unquiet cares, brings to it an unspeakable peace and freedom; this is true simplicity. This state of entire resignation and perpetual acquiescence produces true liberty; and this liberty brings perfect simplicity. The soul which knows no self-seeking, no interested ends, is thoroughly candid; it goes straight forward without hindrance; its path opens daily more and more to 'perfect day,' in proportion as its self-renunciation and its self-forgetfulness increase; and its peace, amid whatever troubles beset it, will be as boundless as the depths of the sea."—*Fénélon.*

"All truly consecrated men learn little by little that what they are consecrated to is not only joy or sorrow, but a Divine idea and a profound obedience, which can find their full outward expression not in joy, and not in sorrow, but in the mysterious and inseparable mingling of the two. . . .

"Under a cloud of circumstances we must walk; but there is behind it that law and that truth which really made the life of Jesus—the law of obedience and the truth of sonship—then for us, too, light shall come through the cloud, and mingling with the darkness make that new condition in which it is best for a man's soul to live, that sweet and strong condition in which both joy and sorrow may have place, but which is greater than either of them—the condition which He called peace."—*Phillips Brooks.*

And if some things I do not ask
In my cup of blessing be,
I would have my spirit filled the more
With grateful love to Thee:
And careful, less to serve Thee much,
Than to please Thee perfectly.

*A. Waring.*

